# Maintenance of empathy levels among first and final year medical students: a cross sectional study

**DOI:** 10.12688/f1000research.2-157.v1

**Published:** 2013-07-16

**Authors:** Areeb Sohail Bangash, Nisreen Feroz Ali, Abdul Haseeb Shehzad, Sobia Haqqi

**Affiliations:** 1 Ziauddin Medical University, Karachi, Pakistan; 2 Department of Psychiatry, Ziauddin Medical University, Karachi, Pakistan

## Abstract

**Objectives:** The purpose of this study was to quantify ****the levels of empathy amongst medical students in the first year and final year of the medical curriculum at a medical university in Karachi, Pakistan.

**Methods:** A cross-sectional study, comprising of participating students in their first year and final year of the medical curriculum at Ziauddin University Medical College, was carried out, using the Empathy Quotient (EQ) scale consisting of 60 questions through a self-administered questionnaire. The results were collected anonymously over a time period of six months from a sample of 171 participants.

**Results** **:** According to our analysis, we found 82.67% of fifth year students and 80.21% of first years showing average or above average levels of empathy. Female mean scores were 42±9.60 while males were 38.7±9.358 (P=0.03). No association was found between empathy and age of the participants (p=0.77).

**Conclusion:** We found no significant difference in the levels of empathy between the first and fifth year medical students. However, it was shown that females exhibited higher levels of empathy than males.

## Introduction

Carl Rogers, one of the founders of the humanist school of psychology, states that empathy is:

" *To sense the patient’s private world as if it were your own but without ever losing the "as if" quality…..to accurately sense the feelings and personal meanings the patient is experiencing and communicating*…" ^1^

Empathy plays a crucial role in the physician-patient therapeutic relationship ^2^. Patient satisfaction and their compliance with treatment improve when their physician understands them better ^3^. In a recent survey, patients of physicians who had scored high in empathy, reported better disease control and prognosis, in comparison to patients of physicians with low empathy scores ^4, 5^.

Empathy is as important for medical students as it is for physicians. Interestingly, there seems to be a decline in empathy levels during medical training as reported by Tavakol *et al.* and Pederson *et al* ^6, 7^. Of the many reasons given to explain this phenomenon, it was reported that, as they progressed through medical school, a number of medical students were starting to become more cynical about life in academia and the medical profession.

The purpose of this study was to quantify empathy levels amongst medical students in the first and the final year of their medical curriculum at a medical university in Karachi, Pakistan.

## Materials and methods

### Ethics statement

Written consent was obtained from each participant. The ethical review committee at Ziauddin University approved this study and the consent procedure.

### Study population

The study participants included 171 out of total 195 medical students from the first and fifth year curriculum of the Medical College at Ziauddin University in Karachi, Pakistan, in 2012. There were a total of 96 students in the first year and 75 students in the fifth year. There were 107 female and 64 male students in the population studied. Students who were not present during the administration of the questionnaire (N=18) and incomplete forms (N=6) were not included in the study. Ziauddin Medical College follows a five-year medical school curriculum with the first three years focused on preclinical study, with limited patient contact, followed by two years of clinical study. The teaching language at the institute is English.

Two cohorts completed the Empathy Quotient Scale questionnaire anonymously, one comprised of first year medical students, the other of fifth year students. The study population included all students who provided complete information in the questionnaire. A list of total students for both the cohorts was extracted from the admissions department at Ziauddin University.

### Instruments used

The medical students completed the Baron-Cohen and Wheelwright Empathy Quotient Scale (EQ), which is a psychometrically validated and reliable instrument for measuring the components of empathy ^8, 9^. The questionnaire used was in its original language (English) and format. The EQ consists of 60 questions, divided into two types: 40 questions judge the empathy of the participant (see Table 1). The remainder are filer questions, which are included to distract the participant from the constant exposure to empathy questions, thus acting as a control.

**Table 1.** Item numbers for empathy assessment and filler questions in the Baron-Cohen and Wheelwright Empathy Quotient Scale.

Question type	Item numbers
Empathy assessment	1, 4, 6, 8, 10, 11,12, 14, 15, 18, 19, 21, 22, 25, 26, 27, 28, 29, 32, 34, 35, 36, 37, 38, 39, 41, 42, 43, 44, 46, 48, 49, 50, 52, 54, 55, 57, 58, 59, 60
Filler	2, 3, 5, 7, 9, 13, 16, 17, 20, 23, 24, 30, 31, 33, 40, 45, 47, 51, 53, 56

Each question had the option of four different responses (strongly agree, slightly agree, slightly disagree and strongly disagree). The "strongly agree" response carried two points while "slightly agree" response scored one point, on the following questions: 1, 6, 19, 22, 25, 26, 35, 36, 37, 38, 41, 42, 43, 44, 52, 54, 55, 57, 58, 59, and 60.

The "strongly disagree" response carried two points while "slightly agree" response score one point, on the following questions: 4, 8, 10, 11, 12, 14, 15, 18, 21, 27, 28, 29, 32, 34, 39, 46, 48, 49, and 50 ^8^.

The participants were asked to provide their age and gender before filling out the EQ. Gender was included because it has been previously reported that female medical students and physicians tend to have more empathy compared to their male counterparts ^10, 11^. As people age they become mature and develop concern for others, which correlates with a gain in empathy hence we included age as a variable ^12^. The age range for first year students was 17–22 years and for fifth year students was 21–26 years.

### Study design/procedure

One author distributed the self-administered questionnaires among the medical students between December 2011 and February 2012. Participation was voluntary and students were assured the responses were confidential. Informed consent was obtained from participants after explaining the purpose of the survey to them. The surveys were conducted immediately after a scheduled lecture, where attendance was compulsory for all medical students within the same cohort. Each student was given ample time to fill the questionnaire; students who were in a hurry were allowed to take the questionnaire and complete it at their own convenience and return it.

Participants who failed to complete or return the administered survey were defined as a non-responder. Baron-Cohen and Wheelwright define a score of 33 or more, out of a total of 80, as adequate empathy levels ^8^. Individuals who scored below 33 were defined as "class 1", they had a lower than average ability to understand and respond to other people’s feelings. Students who scored 33 or more were divided into 3 categories according to their scores. A score between 33–52 was defined as "class 2", where participants showed an average ability to understand and respond to others feelings. A score between 53–63 was defined as "class 3", where the students showed an above average ability to understand and respond to another person’s feelings. A score between 64–80 was defined as "class 4", where the responder showed a very high ability to understand and respond to the feelings of others.

### Statistical analyses

All computation was done using SPSS (version 20). For numeric data mean and standard deviation was used. For categorical data, we used percentages and frequencies. An independent t-test was applied to assess differences between the means of gender and the year of study. The measure of association between age and score was calculated using coefficient of correlation. A chi-square test was applied between the score cohorts, year of study and age group. A P-value lower than 0.05 was deemed significant.

## Results

Of the 177 surveys distributed, 171 were completed and returned. The responders represent 88% of the total body of students in first and fifth year of Ziauddin Medical College.

**Figure 1.**
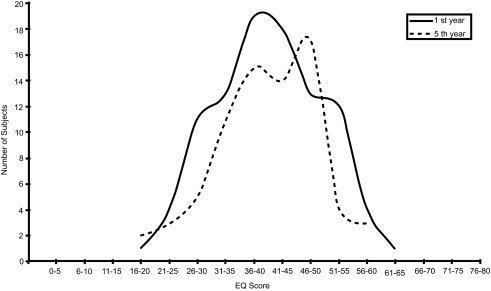
Empathy Quotient **(EQ)** scores in first and final year medical students. EQ scores were not significantly different between year groups.

Figure 1 displays the distribution of EQ scores in the two groups. A comparative analysis revealed that the mean scores for fifth year students (40.85 ± 9.833) were almost equivalent to the first year students (40.70 ± 9.497).

Our analysis of the EQ score for first years revealed that 67.7% of the students had class 2 scores while the remaining 19.8% and 12.5% students scored within classes 1 and 3, respectively. Similarly, the majority of fifth years had class 2 scores (73.3%) while the remaining students obtained class 1, 3 and 4 scores (18.7%, 6.7% and 1.3%), respectively. However, a non-significant association was found between the year of study with EQ scores (P=0.401), as shown in Table 2.

**Table 2.** Association between year of study with empathy class.

Class	1st year N (%)	5th year N (%)	Total
Class I	19 (19.79)	14 (18.67)	33
Class II	65 (67.71)	55 (73.33)	120
Class III	12 (12.5)	5 (6.66)	17
Class IV	0 (0)	1 (1.33)	1
Total	96 (100)	75 (100)	171

We found no association between age and EQ scores (P=0.77).

Table 3 shows the empathy score classification according to gender in both years. According to the data, females had a higher mean score (85%) compared to their male counter parts (75%) when both year groups were combined. These results were also consistent with our findings between sexes within each year group. Female mean scores were 42 ± 9.60 while males were 38.7 ± 9.358 (P=0.03).

**Table 3.** Comparison between the Empathy Quotient score classes with respect to gender.

Gender	Class I	Class II	Class III	Class IV	Total
Male	26.6%	65.6%	7.8%	0%	100%
Female	15%	72.9%	11.2%	0.9%	100%

Empathy quotient scores of 1st and 5th year medical students at a medical university in Pakistan. SPSS data of first year and final year medical students at a medical university in Karachi, Pakistan, with their age, gender, Baron-Cohen and Wheelwright empathy quotient (EQ) score and EQ class (class 1, EQ< 33; class 2, 33-52; class 3, 53-63; class 4, 64-80).

## Discussion

To the best of our knowledge, our study is one of the first to assess empathy amongst medical students in Pakistan. Our results display novel findings, with adequate empathy levels in both first year and final year medical students. Contrary to various studies, which state that empathy decreases as the level of medical education increases ^5, 6^, in our study students in their final year had adequate empathy levels similar to students in first year. This is in support of results obtained in previous Japanese and Korean studies ^13, 14^. Morling and Lamoreaux ^15^ have reported that Asians have more ‘collectivistic and less individualistic social cultures’ than Westerners, and a possible parallel can be drawn between our results and those seen in other culturally similar Asian countries such as Japan and Korea.

The empathy scores recorded amongst senior medical students in our study could also be a result of cohort effects. The emphasis placed during medical training o medical ethics, a considerate attitude towards the patient’s wellbeing and a concentration on a patient centered approach may increase empathy. Furthermore, after the second year of medical school, there is constant patient exposure where students are required to learn history-taking skills and regular examinations that build the student’s professional attitude and approach to gaining patient cooperation, factors that may enhance student empathy. Subjects such as behavioral sciences and medical ethics are taught in the third year of medicine; integrating behavioral sciences at the undergraduate levels can help doctors-in-training to have a better understanding of behavioral issues in clinical settings later on ^16^. Hence, the training obtained throughout medical school may persuade students to implement a sympathetic manner in their interpersonal relationships with patients. It is also worth noting that in Pakistan a great deal of emphasis is placed on apt history taking, focused clinical examinations and particularly minimal lab tests to arrive at a diagnosis. The majority of the population is underprivileged and cannot afford the expense of treatment let alone costly diagnostic tests. Hence, the lack of ‘computer based diagnostic and therapeutic technology’ ^17^ enables physicians and the students shadowing them to rely mainly on good patient interaction and clinical expertise.

In contrast, the literature often shows a positive association of empathy with respect to age. The younger year groups may be less exposed to clinical situations and may hold more idealistic views when starting out in their medical education. Stressors such as academic performance, long work hours ^18– 26^, lack of sleep and subsequent increases in responsibility with age are all contributory factors to a decrease in empathy ^12, 27, 28^. It has been suggested by Rosenfield and Jones ^29^ that a high emphasis is placed on the student’s ability to objectively assess the patient and to maintain a professional and neutral approach. This may all contribute to a decrease in empathy with over the course of medical training.

Through our results, it was also found that females are more empathic than males in both year groups, a finding that is consistent with many international studies ^30– 33^.

Women show a greater understanding of the emotional support that the patient may need and generally tend to give a higher significance to developing inter-personal relationships with patients ^34– 37^, whereas men tend to assign greater significance to authority, independence and control ^38^.

Many reasons have been cited for greater empathy levels in females, including the suggestion that women have evolved to be more gentle and compassionate towards their offspring than their male counterparts ^30^, and hence demonstrate better communication skills and a higher level of understanding towards their offspring. A possible connection can be drawn here, as offspring and patients both require care.

There were several limitations to our research. Due to the cross-sectional nature of our study, we were only able to examine empathy levels in year 1 and year 5. It would be of interest to measure how empathy varies each year throughout the five years of medical school using a prospective longitudinal study design. Secondly, it may also be that the candidates own self-perception influences his/her choices while filling out the questionnaires and this may vary from the actual behavior that is implemented in their everyday interactions. Thirdly, our study only focuses on students attending a private medical school in Pakistan. The results cannot be generalized for all medical colleges and a greater coverage of different medical schools and a larger study population are needed to validate our results further. It is also important to note that other characteristics such as cultural backgrounds and specialty preferences can have an impact on empathy levels; previous research has shown that students opting for ‘people oriented specialties’ show higher empathy levels than those who prefer ‘technology oriented specialties’ ^17^.

The strengths of our study include an appropriate sample size, which represents 88% of the sample population. Our research is the first of its kind in Pakistan and assesses multiple variables such as age, gender and year of education.

## Recommendations

It would be valuable to carry out a prospective study where students are followed annually from the beginning of first year until graduation, to give a true representation of change in empathy levels. Other variables such as cultural backgrounds and specialty preferences should be noted.

## Conclusion

Medicine is a field where interpersonal skills and concern for others are of key importance, for it is a field whose very core is based on service to humanity. It is thus essential that empathy should be nurtured in medical students rather than eroded away with time and clinical exposure. It was surprising to see that a large majority of both first years and fifth years maintained adequate empathy levels despite no significant emphasis placed on the matter throughout the medical curriculum. Gender differences were significant and further research needs to be carried out to determine reasons for this. Our research paves the way for further research to be carried out with Pakistani medical students, which may further justify our results and identify additional influencing variables.
