# Supplementary material for: Maintenance of empathy levels among first and final year medical students: a cross sectional study
Source: F1000Res. 2013 Jul 16;2:157. [Version 1] doi: 10.12688/f1000research.2-157.v1 (PMC3782343; doi:10.12688/f1000research.2-157.v1)
Supplement: Peer review history

# Peer review: Referee response for version 1

**Published:** 2013-07-29

**Authors:** Iman Hegazi

**Affiliations:** 1 Medical Education Unit, University of Western Sydney, NSW, Australia

I have read the manuscript with great interest. Empathy will remain an area of high importance for medical education and, despite being extensively studied, its research should still be encouraged world-wide.This is well-conducted research and the authors have succeeded in explaining the data clearly and concisely. The abstract is concise and provides an adequate summary of the article. The article is clearly written and well constructed.I recommend only a few minor changes:I believe that the current title may be somewhat misleading as the word “maintenance” implies a more longitudinal study, or a study across all cohorts from first to final year, whereas this research is more of a comparative cross-sectional study between first and final year medical students. I recommend that a more appropriate title be used.The methodological rigor is adequate with only a few pointers:Since the EQ test was not included in the article, I do not see any need for paragraphs 5 and 6 in the “Materials and methods” section. These paragraphs did not help explain the EQ and those who are interested in understanding the EQ test can easily refer to the reference. Similarly, table 1 does not add any information and can be removed.In the statistical analyses section, it was mentioned that the t-test and chi-square test were used. The values of these tests along with the p-values should be included in the text and in tables 2 and 3.The discussion and conclusion appear to be sensible and balanced. It would have been interesting to tell us about the selection process that medical students have to go through before being accepted in this private medical school.Overall, this is a well-written and interesting article.

I have read this submission. I believe that I have an appropriate level of expertise to confirm that it is of an acceptable scientific standard.

# Peer review: Referee response for version 1

**Published:** 2013-07-25

**Authors:** Pooria Foroushani

**Affiliations:** 1 Faculty of Medicine, University of New South Wales, Sydney, Australia

The article has an appropriate title, and the abstract provides adequate information about the study. The paper is well written, containing clear and balanced data about the study and related literature. Bangash et al have used a standard questionnaire to compare empathy level of first and five year medical students. They had a high response rate and have found that empathy level remains at the high level of 80%. The authors have referred to the limitation of their methodology. They have argued that this finding is different from Westerners’ studies and is similar to the results of researches in other Asian countries, such as Japan and Korea. This is an interesting topic for further explorations. As Bangash et al have also recommended, it will be interesting to undertake prospective longitudinal studies and also to explore to the relationship between self-reported empathy with actual interactions with patients.

I have read this submission. I believe that I have an appropriate level of expertise to confirm that it is of an acceptable scientific standard.

# Peer review: Referee response for version 1

**Published:** 2013-07-19

**Authors:** Kevin J Black

**Affiliations:** 1 Washington University in St. Louis, St Louis, MO, USA

The authors surveyed first- and fifth-year medical students in a private medical school in Pakistan with the goal of quantifying empathy according to a standard questionnaire. The hypothesis was that medical students' empathy declines during training. 

A high survey return rate was achieved (88%). Women scored higher on empathy than men, which fits the results of other studies. However, there was no significant difference in empathy scores between beginning and senior medical students whether viewed as a mean or as a distribution between typical, low, and high score categories. 

The authors discuss possible explanations for their results appropriately and do not go beyond the data. They note the primary limitations: the cross-sectional approach and the focus on one school. 

The authors are impressed that ~80% of the students scored in the normal to above normal empathy ranges. By contrast, I was surprised that 19-20% of the students scored in the below-normal range. After all, these are people entering a profession devoted to taking care of others! I am not aware of whether other studies of empathy among physicians or trainees show higher or lower rates of empathy than in the general population, but if so, a mention of those results in Discussion would add interest.

Overall, this is an interesting tidbit of information about medical training, presented clearly and without exaggeration.

I have read this submission. I believe that I have an appropriate level of expertise to confirm that it is of an acceptable scientific standard.
